# Retrospective Correction of Physiological Noise in DTI Using an Extended Tensor Model and Peripheral Measurements

**DOI:** 10.1002/mrm.24467

**Published:** 2012-08-30

**Authors:** Siawoosh Mohammadi, Chloe Hutton, Zoltan Nagy, Oliver Josephs, Nikolaus Weiskopf

**Affiliations:** Wellcome Trust Centre for Neuroimaging, UCL Institute of Neurology, University College LondonLondon, UK

**Keywords:** DTI, robust fitting, physiological noise, cardiac pulsation and respiration artefacts, fractional anisotropy

## Abstract

Diffusion tensor imaging is widely used in research and clinical applications, but this modality is highly sensitive to artefacts. We developed an easy-to-implement extension of the original diffusion tensor model to account for physiological noise in diffusion tensor imaging using measures of peripheral physiology (pulse and respiration), the so-called extended tensor model. Within the framework of the extended tensor model two types of regressors, which respectively modeled small (linear) and strong (nonlinear) variations in the diffusion signal, were derived from peripheral measures. We tested the performance of four extended tensor models with different physiological noise regressors on nongated and gated diffusion tensor imaging data, and compared it to an established data-driven robust fitting method. In the brainstem and cerebellum the extended tensor models reduced the noise in the tensor-fit by up to 23% in accordance with previous studies on physiological noise. The extended tensor model addresses both large-amplitude outliers and small-amplitude signal-changes. The framework of the extended tensor model also facilitates further investigation into physiological noise in diffusion tensor imaging. The proposed extended tensor model can be readily combined with other artefact correction methods such as robust fitting and eddy current correction.

## INTRODUCTION

Diffusion tensor imaging (DTI) allows for noninvasive imaging of water diffusion (1–4), which is an important marker for brain anatomy and physiology. Because of its sensitivity to microstructural and physiological changes, DTI has not only become a wide spread imaging method in neuroscience research (5–7) but also an essential diagnostic tool after stroke or in detecting neurodegenerative disease (8–14).

Despite its ubiquitous use in research and clinical application, artefacts can occur during data acquisition, which reduce the reliability of DTI data (15–17). One group of artefacts that is still not sufficiently addressed in diffusion MRI results from contamination introduced by normal human physiological processes such as breathing and heartbeat. The diffusion sensitization makes the diffusion MRI sequence susceptible to any kind of movements (18–21). As a result, not only the desired microscopic Brownian movement of water molecules affects the diffusion signal but also the undesired macroscopic movement of brain tissue originating, e.g., from the human physiological processes. In the extreme, the latter kind of movement can even lead to signal-loss (18,22,23).

Physiological artefacts can be reduced by confining the acquisition to the relatively quiet diastolic period of the cardiac cycle using cardiac triggering (22,23). However, triggering increases the scanning time. Recently, new promising data-driven approaches (here denoted as robust fitting model) were suggested to retrospectively detect artefacts associated with physiological noise directly from the DTI data (15,17,24,25). Estimating physiological artefacts directly from the data, however, can be complicated in the presence of other noise sources. Examples of other noise sources are residual vibration and eddy current artefacts, which could not be fully corrected by the respective retrospective correction methods (19,26–28). An alternative method to estimate and reduce the effect of physiological artefacts is known from investigations into fMRI, where additional regressors, based on estimates of head and peripheral measurements of cardiac pulsation and respiration, are included in the fMRI general linear model (29–37).

We translate and adapt this approach to DTI with the goal to introduce an easy-to-implement, retrospective correction method for physiological artefacts using peripheral measurements. For this, we propose a novel extended diffusion tensor model that incorporates regressors to explain physiological effects. We applied variations of our physiological noise correction models to nongated and gated DTI data, and compared the correction results with the results of a data-driven robust fitting model of Zwiers (38).

## METHODS

### Theory

In the original diffusion tensor representation of Basser et al. (39), the diffusion-weighted (DW) signal *S*_*i*_ that is generated by applying a diffusion gradient along the *i*th direction is given by:

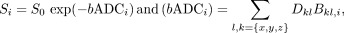
[1]
where *S*_0_ is the non-DW signal, *b* summarizes the extent of diffusion sensitization as described by Mattiello et al. (40), and the apparent diffusion coefficient, ADC_*i*_, is related to the diffusion tensor, **D**, via the **B**_***i***_ matrix [Basser et al. (39)]. The elements of the **B** matrix 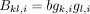 are functions of the diffusion gradient vector components ***g***_*i*_ = (*g_x,i_ g_y,i_ g_z,i_*), with 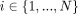, and *N* equals the number of diffusion gradient directions. The diffusion tensor, **D**, can be calculated from the ADCs in Eq. [1] using, e.g., a least-square fit:

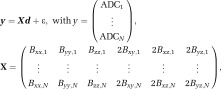
[2]
where **X** is the tensor design matrix, i.e., an *N* × 6 matrix that is constructed from the **B** matrix elements (39), 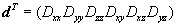, and ε is the tensor-fit error.

To account for the effects of physiological noise within the diffusion signal, we assumed that the noise can be modeled as a time-dependent multiplicative term 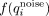 with respect to the DW signal:

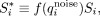
[3]
where 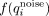 is a dimensionless, time-dependent function of the noise varying with diffusion gradient directions, ***g***_*i*_. Because the diffusion gradients are applied sequentially, each diffusion gradient direction, ***g***_*i*_, can be represented by a point on a time axis 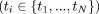, counting in steps of volume repetition time, 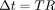. It follows from Eq. [3] that the perturbed ADC 
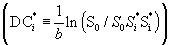
 is:

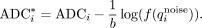
[4]

To calculate the corrected diffusion tensor, **D**^cor^, from the perturbed ADC in Eq. [4], the columns of **X** must be extended by additional regressors, which account for the perturbations:

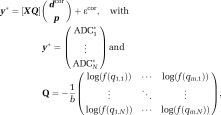
[5]

In Eq. [5], the elements of the diffusion tensor are summarized as a vector ***d***^cor^, **Q** is given by an *N* × *m* matrix including physiological noise regressors (*m* regressors with the length *N*), the components of the ***p*** vector are the weighting factors of each regressor, and ε^cor^ is the error of the new tensor-model fit in Eq. [5].

In the following, the columns of **X** are denoted as tensor regressors and the columns of the **Q** matrix as physiological noise regressors or in short noise regressors. The model in Eq. [5] for estimating the tensor and noise components will be named the extended tensor model. In the case of using no noise regressors, i.e., setting the **Q** matrix to zero, the extended tensor model reduces to the standard tensor model of Basser et al. (39).

#### Noise Model with Linear Regressors

If the noise is assumed to vary linearly with respect to the regressor *q*_*i*_, Eq. [3] can be written as:

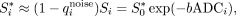
[6]
where 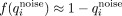 and 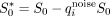.

In Eq. [6], the noise has been modeled by a time-dependent additive term with respect to the *T*_2_-weighted signal, which is similar to the assumption that has been successfully used to model physiological noise in fMRI (41).

For this model, the linearized Q-matrix in Eq. [5] becomes:

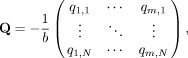
[7]
where the following approximation has been used 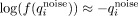. Note that the same formalism as derived in Eq. [6] can also be used to model linear noise, which is directly related to the diffusion tensor (by replacing 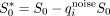 with 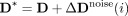 in Eq. [6]).

#### Noise Model with Nonlinear Regressors

**Fig 1:**
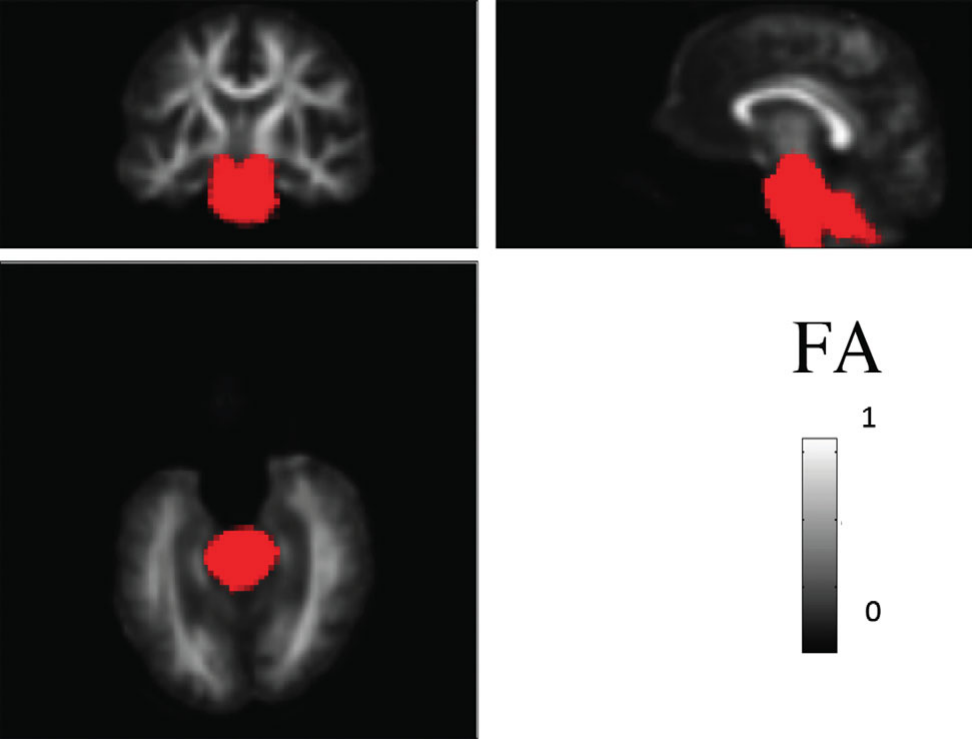
ROI in the lower basal area (in red) is projected on the customized FA template. The choice of this region was motivated by results of previous investigation into physiological noise in DTI data, which indicated an increased level of noise in this area (17). [Color figure can be viewed in the online issue, which is available at wileyonlinelibrary.com.]

An alternative noise model is based on observations in previous diffusion MRI studies on cardiac pulsation artefacts (22,23,42). It has been suggested that the bias in the diffusion signal can be explained by a shift of the *k*-space centre that depends on the tissue motion induced by cardiac pulsation and the amplitude of the diffusion gradients vector in the *z*-direction (42). This signal-bias depends also on the image reconstruction (19) and in particular on the *k*-space apodization function (28,43). We modeled the apodization function by a Gaussian function multiplied with a Heaviside function:

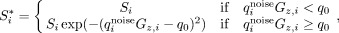
[8]
where *G*_*z*,*i*_ is the *z*-component of the *i*th diffusion gradient vector, *q*_0_ is a constant which depends on the image reconstruction details (i.e., on shape of the apodization function). We empirically determined that *q*_0_ = 0.5 results in a good detection of the physiological noise artefacts within the basal region of interest (ROI; for definition of the basal ROI see Fig. 1) for our acquisition protocols. Note that the value of *q*_0_ might change for different types of diffusion acquisition protocols.

For this model, the Q-matrix in Eq. [5] becomes:

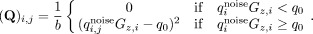
[9]

Note that this approximation does not include cross-terms of the form 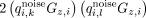 (with 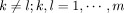 and 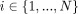). Including them would increase the condition number of the extended diffusion tensor substantially, which can deteriorate the tensor fit (see methodological considerations). In simulations (data not shown), we observed that the effect of the perturbations could be largely corrected even if the cross-terms were neglected.

### Subjects

Six healthy adult volunteers (one female, five male) participated in the study approved by the local ethics committee after giving written informed consent.

### Data Acquisition and Preprocessing

Experiments were performed on a TIM Trio 3 T scanner (Siemens Healthcare, Erlangen, Germany) operated with an RF body transmit coil and a 32-channel receive-only head coil. DTI data were acquired with an in-house developed DTI sequence (44,45) based on the twice-refocused spin echo diffusion scheme of (46,47) and using the following parameters: 60 DW images with spherically distributed diffusion-gradient directions (48) (*b* = 700 s/mm^2^), six low-DW images (*b* = 100 s/mm^2^), 96 × 96 acquisition matrix, 256 mm field of view, 50 slices, 2.7-mm isotropic resolution, asymmetric echo shifted forward by 25 phase-encoding lines, echo time of TE = 81 ms.

For each subject two sets of DTI data were acquired, using a gated (DTI_g_) and normal, nongated (DTI_n_) diffusion sequence. The gated data were acquired in blocks of three slices per cardiac cycle. The total volume repetition time of the cardiac gated DTI data depended on the heart rate but was 17 s on average. The repetition time of the nongated DTI data was 8.5 s. Because of technical problems one gated DTI dataset was discarded, yielding five gated and six nongated DTI data sets.

During scanning sessions peripheral measurements of the subject’s pulse and breathing were recorded together with scanner slice synchronization pulses [similar to Hutton et al. (29)] using the Spike2 data acquisition system (Cambridge Electronic Design Limited, Cambridge, UK). The cardiac pulse signal was measured using an MRI compatible pulse oximeter (Model 8600 F0, Nonin Medical, Inc. Plymouth, MN) attached to the subject’s finger. The respiratory signal, thoracic movement, was monitored using a pneumatic belt positioned around the abdomen close to the diaphragm.

The DTI datasets were preprocessed by correcting for motion and affine whole-brain eddy current image distortions [Mohammadi et al. (26)]. After preprocessing, the ADCs were estimated using the standard tensor model of Basser et al. (39). Then, the models in Table 1 were used to estimate the diffusion tensor, the tensor-fit error (ε), and the fractional anisotropy (FA) as defined in Ref. 4. The data-driven robust fitting model (vi) was based on the method of Zwiers (38). The basic idea of the robust fitting method is to down-weight outliers in the data that fall far outside the expected spread of the nonoutlier residuals *C*, which is proportional to the median of the tensor-fit error 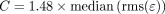 (49,50). Note that our in-house implementation of the robust-fitting approach did not correct for severe subject-motion artefacts (i.e., signal-loss across whole slices), because we did not observe those kinds of artefacts in our data. All analysis steps were performed using SPM8 [http://www.fil.ion.ucl.ac.uk/spm, (51)], the EC and motion correction SPM toolbox [http://www.fil.ion.ucl.ac.uk/spm/ext, (26)], and in-house software written in MATLAB (version 7.11.0; Mathworks, Natick, MA).

**Table 1:** Models for Diffusion Tensor Estimation

Model (*m*)	Tensor estimation	Linear noise regressors (number)	Nonlinear regressors (number)	Total number of regressors *p*^(*m*)^
(i)	Standard tensor model	n.a.	n.a.	6
(ii)	Extended tensor model	Cardiac phase (4)	None	10
(iii)	Extended tensor model	Cardiac and respiratory phase (8)	None	14
(iv)	Extended tensor model	None	Cardiac phase (2)	8
(v)	Extended tensor model	Respiratory phase (4)	Cardiac phase (2)	12
(vi)	Robust fitting	n.a.	n.a.	6

The different models that were used to estimate the diffusion tensor are depicted. The total number of regressors *p*^(*m*)^ consists of the six tensor regressors and *m* noise regressors. Note that although the total number of regressors is six for the robust fitting model the effective degrees of freedom can be reduced by more than 6 because of the down-weighting of outliers.

### Estimating the Noise

To estimate the noise regressors, the peripheral measurements (thoracic movement representing respiration and pulse) were preprocessed and the cardiac and respiratory phases were calculated using an in-house developed Matlab toolbox (29). This toolbox uses models for cardiac and respiratory phases and their aliased harmonics which are based on RETROICOR (30) and similar, earlier methods (52). Physiological noise regressors were constructed from basis sets of sine and cosine Fourier series components extending to the second harmonic for both the cardiac and the respiratory phase. The total number of regressors included in each extended tensor model are summarized in Table 1. The high temporal resolution of the peripheral measurements of the subject’s pulse and breathing allowed us to calculate slice-dependent respiratory and cardiac phase regressors. Because of the slice-dependent noise regressors the **Q** matrix will change from slice-to-slice, resulting in a slice-dependent design matrix in Eq. [5].

### Assessment of Physiological Noise Correction

#### Spatial Characteristics of Noise Models

To visualize the spatial characteristics of the noise correction models, the root-mean-square (rms) map of the tensor-fit errors was calculated for each model as listed in Table 1. To this end, the difference image between the adjusted rms-tensor-fit errors of the correction models (ii)–(vi) and the standard model (i) was calculated: 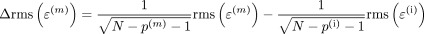, where *N* is the number of DW directions and *p*^(*m*)^ is the total number of regressors used in the tensor model *m* (see Table 1.). Note that for the robust fitting model, the effective number of DW directions might differ from *N* = 60 and vary from voxel-to-voxel. The effective number of DW directions is given by the number of directions for which the residual error is smaller than the expected spread of the nonoutlier residuals *C*.

#### ROI Analysis to Assess Performance of the Correction Models

To compare the performance of the different models (see Table 1), ROI analyses were performed. To this end, a customized FA template and low-DW contrast template (*b* = 0 template) was constructed in the subjects' native space using the FA-VBS normalization toolbox [http://www.fil.ion.ucl.ac.uk/spm/ext/, (53)]. The first *b* = 0 image of each subject from both datasets, DTI_g_ and DTI_n_, were registered to the *b* = 0 template using a 12-parametric affine registration. Based on the FA template a ROI mask was constructed in the lower region of the brainstem and cerebellum (see Fig. 1).

The reduction in the tensor-fit error with respect to the standard tensor model (i) was calculated within the brainstem ROI (see Fig. 1) and within the whole brain using the models *m* = (ii)–(vi):

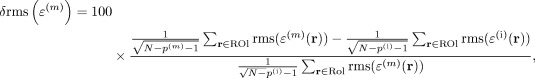
[10]
where *N* is the number of DW directions and *p*^(*m*)^ is the total number of regressors used in the tensor models *m* (see Table 1).

#### Effect of gating

To investigate the effect of gating and its interaction with the correction models (see Table 1), the histogram of the adjusted rms-tensor-fit error was calculated within the whole brain for the gated and nongated DTI data. To quantify the distribution of the rms-tensor-fit error, a Rician distribution was fitted to the normalized histogram. The mode of the distribution was used to quantify the effect of the different models on the rms-tensor-fit error.

#### Spatial Characteristics of the Correction Models with Respect to the FA

The spatial characteristics of the correction models with respect to the FA were investigated. For this purpose, the FA difference maps between the standard model and the noise correction models were calculated 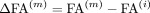 (*m* = (ii)–(vi), see Table 1).

#### Performance of the Correction Models with Respect to the FA

The performance of the correction model was assessed in two steps. First, the magnitude of the voxel-wise difference in the FA values before and after correction was calculated within the brainstem ROI (Fig. 1) for models *m* = (ii)–(vi):

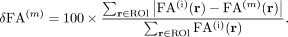
[11]

In the second analysis, we assessed whether the extended tensor models (ii)–(v) increased or reduced the difference in FA relative to the results of the robust fitting method (vi). To this end, the normalized cross-correlation between the 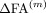 maps of the extended tensor models [models *m* = (ii)–(v)] and the robust fitting model 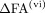 was calculated:

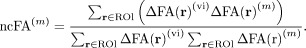
[12]

Note, that we implicitly used the robust-fitting model as a reference. However, it should be regarded a silver standard rather than a gold standard, because it only corrects outliers but not small amplitude perturbations [see, e.g., (38), Fig. 4]. Furthermore, it corrects not only physiological-noise-induced outliers but also outliers of different origin, such as eddy currents (26), vibration (19,28), and subject motion (26,38).

## RESULTS

### Spatial Characteristics of Noise Models

**Fig 2:**
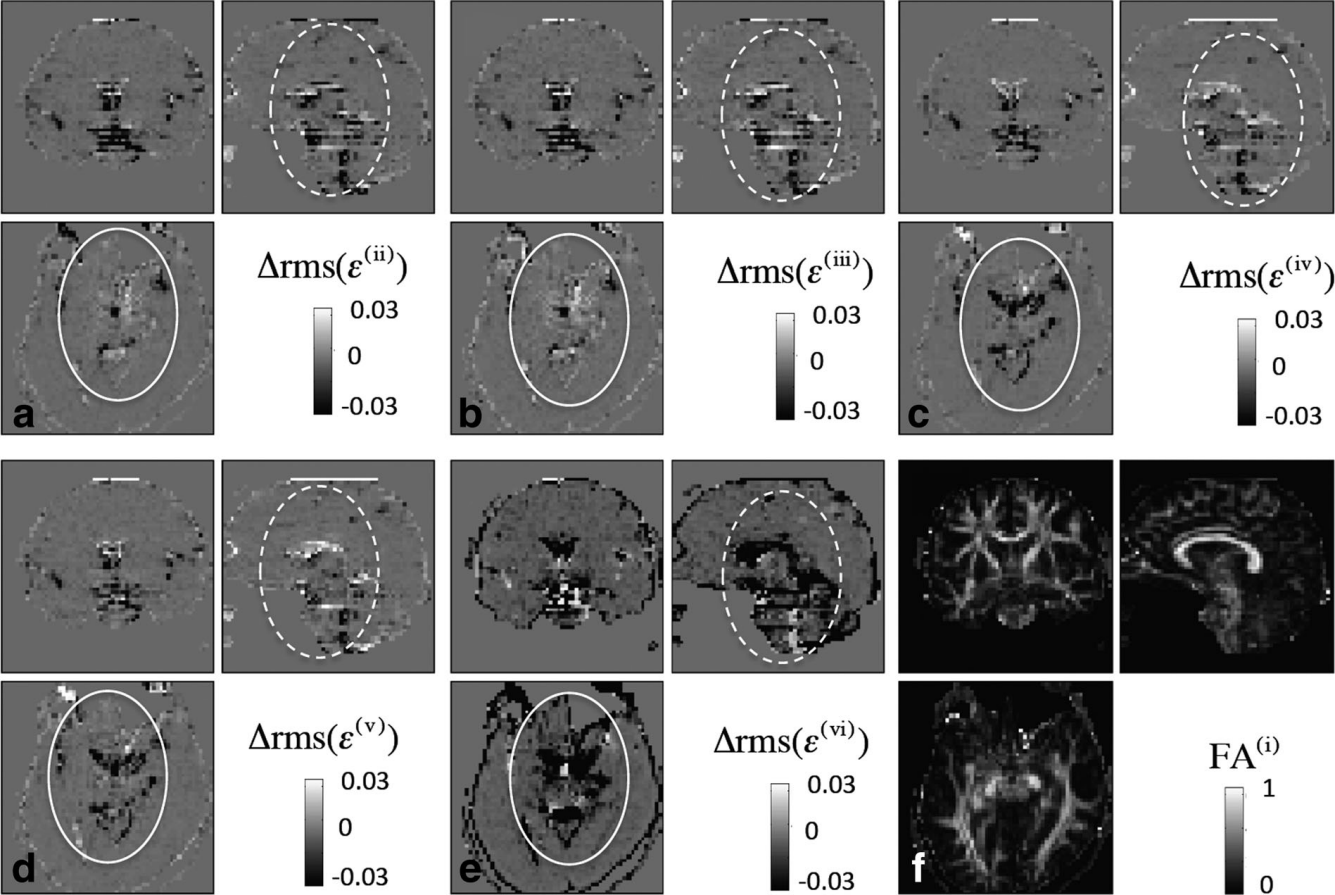
The spatial noise characteristics of the nongated (DTI_n_) data using the extended tensor models (a and b) with linear regressors [model (ii) and (iii)], (c and d) with nonlinear regressors [model (iv) and (v)], and (e) the robust fitting model (vi) (for an overview of the models, see Table 1). To determine how much noise can be explained by each model, the difference map of the adjusted rms of the tensor-fit errors before and after correction [Δrms(ε^(*m*)^), *m* = (ii)–(vi)] is depicted for one representative subject. For easier anatomical localization the corresponding FA image is depicted in (f). The effect of the noise correction was most pronounced in the basal brain regions and the brainstem (dashed circle). The robust fitting correction performs best [model (v)]. The spatial noise characteristics differ between the linear-regressor [model (ii)–(iii)] and the nonlinear-regressor [model (iv)–(v)] extended tensor models (solid circles). Note that the noise correction sometimes increases the adjusted tensor error due to a reduction in degrees of freedom.

**Fig 3:**
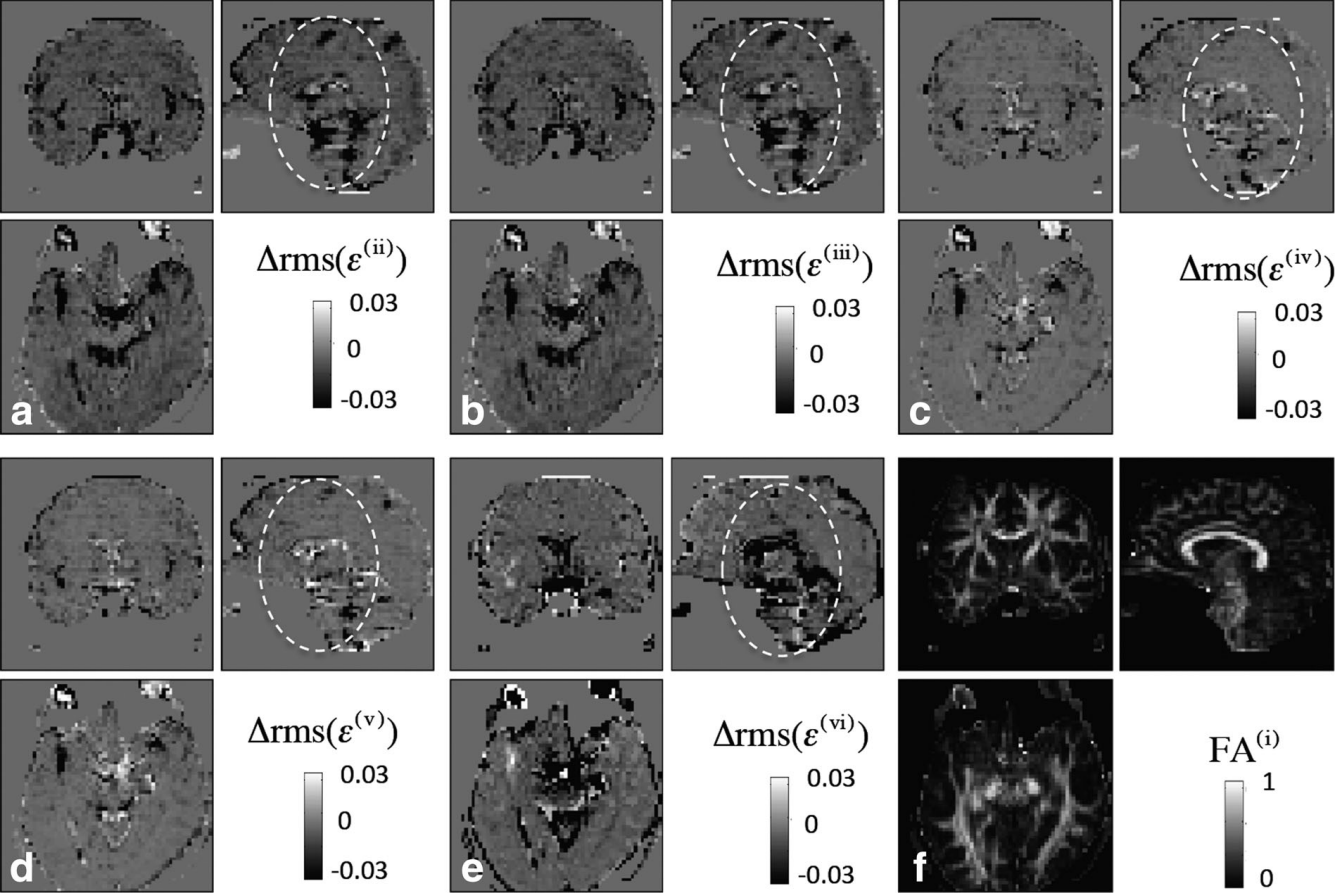
Spatial noise characteristics of the gated data (DTI_g_) using the extended tensor models (a and b) with linear regressors [model (ii) and (iii)], (c and d) with nonlinear regressors [model (iv) and (v)], and (e) the robust fitting model (vi). For explanation, see caption of Figure 2. The noise correction is most pronounced in the basal brain regions and the brainstem (dashed circle). The linear extended tensor models (ii) (a) and (iii) (b) and the robust fitting model (vi) (e) showed the highest performance.

Figures 2 and 3 show the spatial characteristics of the noise correction models (ii)–(vi) in the nongated and gated DTI data respectively using the rms-tensor-fit error. For both datasets, the physiological noise was most apparent in the basal brain areas, cerebellum, at the edge of the cortex, and at boundaries between tissue and cerebral spinal fluid, e.g., next to the ventricles. The noise correction did not always reduce the tensor-fit error. For the nongated DTI data (Fig. 2), the spatial pattern of the linear-regressor models (ii) and (iii) differed from the nonlinear-regressor models (iv) and (v): the corrected noise highlighted by the dashed circle was better explained by linear-regressor models (ii) and (iii), whereas the noise highlighted by the solid circle was better explained by the nonlinear-regressor models (iv) and (v). Altogether, the noise in both highlighted regions in Figure 2 was most effectively corrected using the robust fitting model (vi). For the gated data (Fig. 3), the nonlinear-regressor models (iv) and (v) corrected the least amount of noise over the whole brain, while the other correction models (ii), (iii), and (vi) reduced the noise very effectively.

### ROI Analysis to Assess Performance of the Correction Models

**Fig 4:**
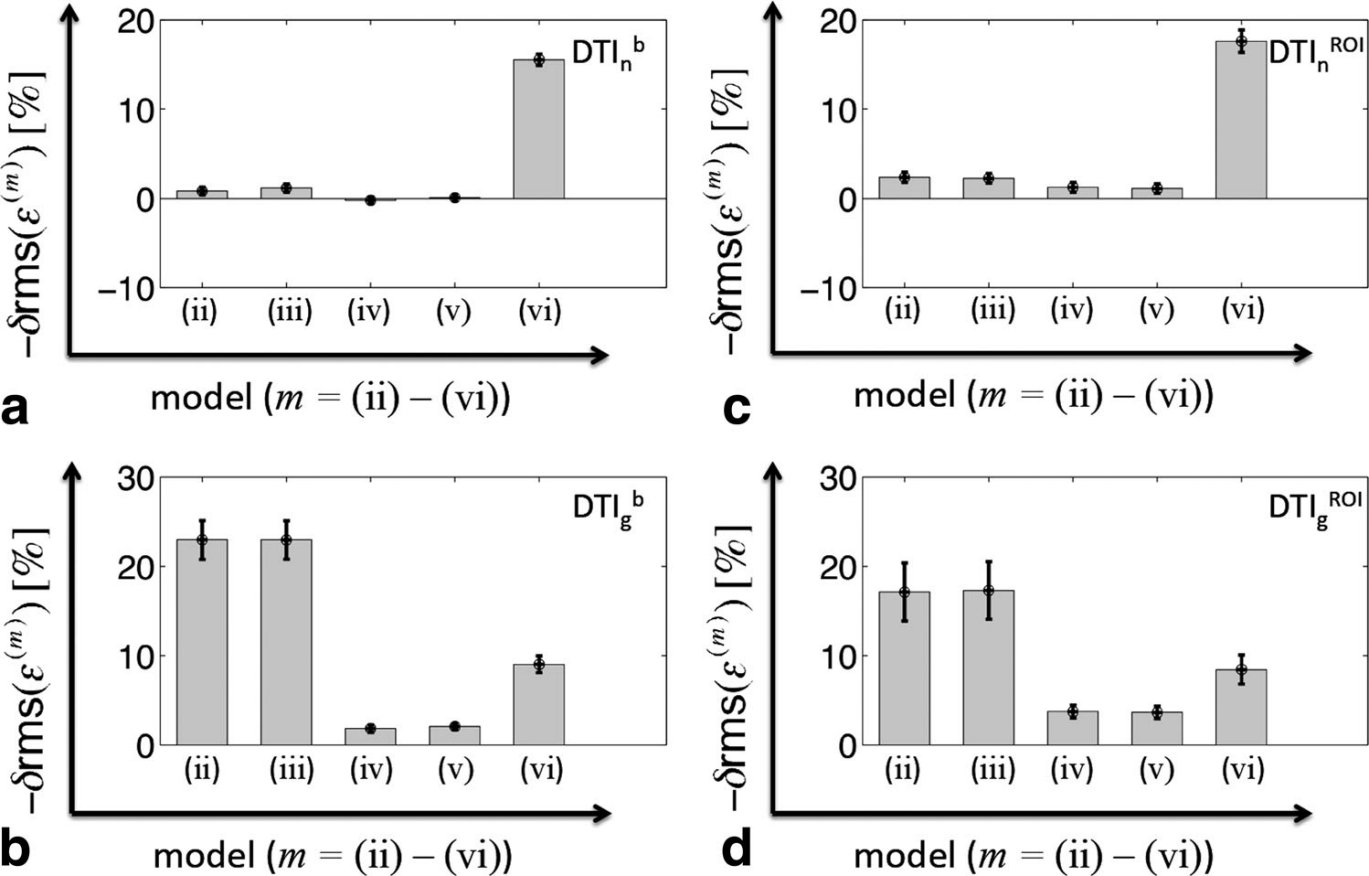
Quantitative comparison of the physiological noise correction within the whole brain (a and b) and the brainstem ROI (c and d) using the extended tensor with linear regressors [model (ii) and (iii)], with nonlinear regressors [model (iv) and (v)], and the robust fitting model (vi) (for an overview of the models, see Table 1). The relative improvement of the adjusted rms of the tensor-fit error [−δrms(ε^(*m*)^), *m* = (ii)–(vi)] with respect to the standard tensor model (i) is depicted. For the nongated data (DTI_n_, top row), the reduction in the tensor-fit error was maximal when the robust fitting model (vi) was used (about 18%). For the gated data (DTI_g_, bottom row), the reduction in the tensor-fit error was maximal for the extended models (ii) and (iii) (about 23%). Note that the negative of δrms(ε^(*m*)^) (Eq. [10]) is depicted, i.e., the reduction of the tensor-fit error is presented as positive percentage value.

Figure 4 summaries the relative performance of the correction models within the whole brain (Fig. 4a,b) and brainstem ROI defined in Figure 1 (Fig. 4c,d). For the nongated data (Fig. 4a,c), the reduction in noise was greatest when the robust fitting model (vi) was used and all models explained more noise within the ROI than over the whole brain. For the gated data (Fig. 4b,d), the linear-regressor models (ii) and (iii) explained more noise than the other models. Moreover, for the gated data the linear-regressor models (ii) and (iii) explained less noise in the brainstem ROI than in the whole brain. No significant additional noise was explained when the respiratory regressors were added [i.e., when using models (iii) and (v)].

### Effect of Gating

**Fig 5:**
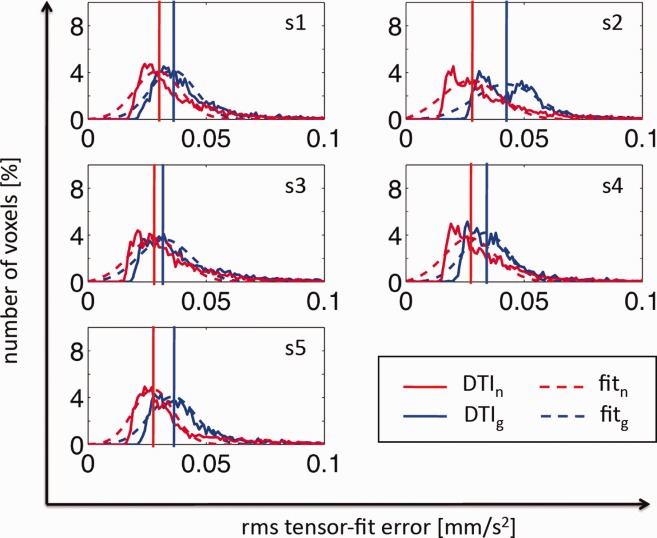
Comparison of the tensor-fit error between gated and nongated DTI data for five subjects (s1–s5). To this end, the histogram of the adjusted rms of the tensor-fit error (adjusted for effective degrees of freedoms) is shown for the gated (blue solid line) and nongated (red solid line) data using the standard tensor model (i). The histogram of the adjusted rms-tensor-fit error was fitted by a Rician distribution function (dashed lines) and the mode of the distribution was identified (vertical lines). Unexpectedly, the tensor-fit error (i.e., the mode of the histogram of the adjusted rms-tensor-fit error) was always smaller for the nongated than for gated data.

**Fig 6:**
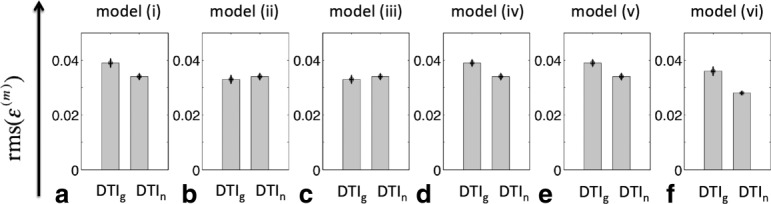
Group comparison of tensor-fit error of gated (DTI_g_) and nongated (DTI_n_) data using (a) the standard tensor model (i), the extended tensor models (b and c) with linear regressors [model (ii) and (iii)], (d and e) with nonlinear regressors [model (iv) and (v)], and (f) the robust fitting model (vi). To this end, first the mode of the Rician fit of the adjusted rms-tensor-fit error histogram was determined for each subject (see Fig. 5). Then, the median and the standard error of the mean of the modes were calculated. The mode of the Rician fit was most similar between gated and nongated data when the correction model (ii) or (iii) was used (b and c). The mode of the Rician fit was smallest when model (vi) was used for the nongated data (f).

Figures 5 and 6 compare the adjusted tensor-fit error between gated and nongated DTI data. Figure 5 shows the histogram of the rms-tensor-fit error for five subjects using the standard tensor model (i). Figure 6 shows a groupwise comparison of the modes of the fitted Rician distributions for the models (i)–(vi). When using the standard tensor model (i), the mode of the Rician distribution was always larger for the gated than for the nongated data (Figs. 5 and 6a). For the nongated data, the largest reduction of the mode of the Rician distribution was achieved when using the robust fitting model (vi) (Fig. 6f). For the gated data, linear-regressor models (ii) and (iii) resulted in the largest reduction (Fig. 6b,c). Moreover, the modes of the Rician distribution were most similar between gated and nongated data when linear-regressor model (ii) or (iii) was used (b and c).

### Spatial Characteristics of the Correction Models with Respect to FA

**Fig 7:**
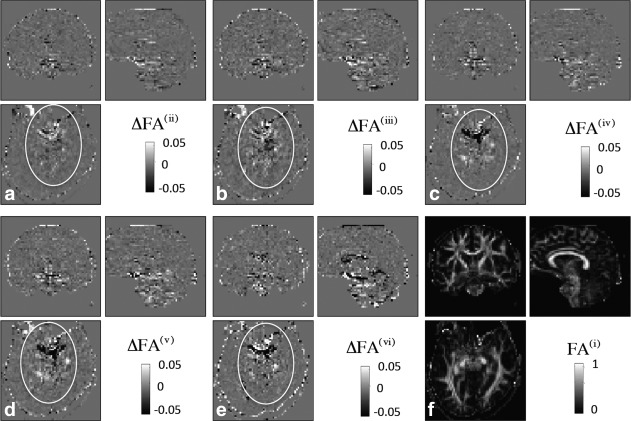
Effect of noise correction models on FA maps for the nongated DTI data using the extended tensor models (a and b) with linear regressors [model (ii) and (iii)], (c and d) with nonlinear regressors [model (iv) and (v)], and (e) the robust fitting model (vi) (for an overview of the models, see Table 1). To this end, FA difference images before (FA^(i)^) and after [FA^(*m*)^, *m* = (ii)–(vi)] correction were calculated (ΔFA^(*m*)^=FA^(*m*)^−FA^(*i*)^) using the same subject as in Figure 2. For easier anatomical localization the corresponding FA image is depicted in (f). Within the highlighted region (solid circle), the FA difference maps obtained from the nonlinear-regressor models [(iv) and (v)] were similar to those from robust-fitting [model (vi)]. No evident similarities were visible between the maps obtained from the linear-regressor models [(ii) and (iii)] and those from the robust fitting model (vi). Note that the highlighted regions in the FA difference maps resemble the same regions that were highlighted in Figure 2 by solid lines.

**Fig 8:**
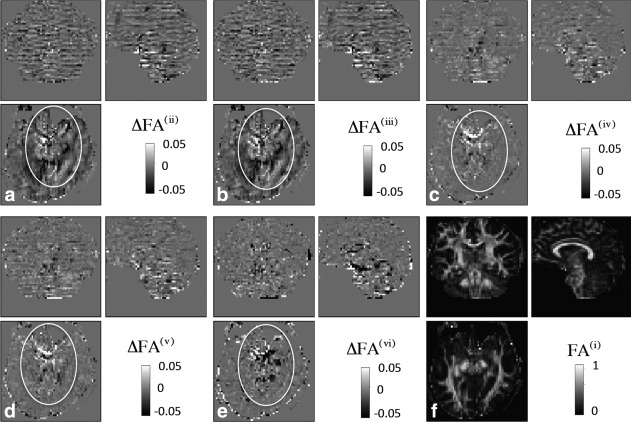
Effect of noise correction models on FA maps for the gated DTI data using the extended tensor models (a and b) with linear regressors [model (ii) and (iii)], (c and d) with nonlinear regressors [model (iv) and (v)], and (e) the robust fitting model (vi). For details see legend of Figure 7. While the correction models (iv)–(vi) mostly affected structures within basal regions (see solid circle), the correction models (ii) and (iii) affected the FA within the white matter over the whole brain. Note that the data were acquired with an interleaved slice acquisition order; this might explain the alternating structure in the ΔFA maps that is particularly visible in models (ii) and (iii), which varied between subjects.

The maps in Figures 7 and 8 show how noise correction changed FA estimates for the nongated and gated DTI data, respectively. For the nongated data (Fig. 7), all correction models mostly affect basal regions of the FA map. The highlighted region in Figure 7 shows a region where the FA differences obtained the nonlinear-regressor models (iv) and (v) resemble those of the robust fitting model (vi). For the gated data (Fig. 8), the nonlinear-regressor models (iv), (v), and the robust fitting model (vi) lead to FA differences within basal regions, whereas the linear-regressor models (ii) and (iii) affect the white matter structures over the whole brain.

### Performance of the Correction Models with Respect to the FA

**Fig 9:**
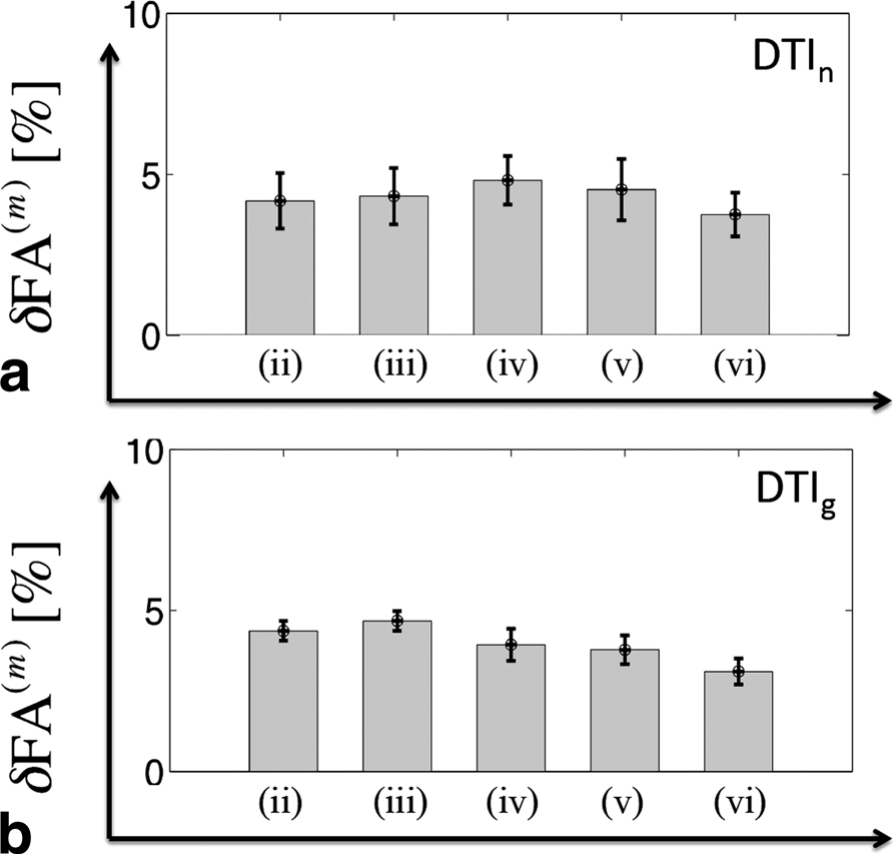
The effect of the correction models (ii)–(vi) within the brainstem ROI (defined in Fig. 1) on the magnitude of the FA image for nongated (a) and gated (b) DTI data. To this end, the relative change (δFA^(*m*)^, Eq. [11]) between the FA before and after correction was calculated when using the extended tensor models with linear regressors [model (ii) and (iii)], with nonlinear regressors [model (iv) and (v)] and the robust fitting model (vi). For an overview of the models, see Table 1. The correction models changed the FA by 3–5%. The effect on the FA was minimal when the robust fitting model (vi) was used on gated DTI data.

Figure 9 shows the effect of the correction models (ii)–(vi) (see Table 1) on the FA averaged over the brainstem ROI depicted in Figure 1. The greatest FA difference was obtained for the gated data when using the linear-regressor model (iii) and for the nongated data when using the nonlinear-regressor model (iv). The smallest FA difference was obtained for the robust fitting model (vi) for both the gated and nongated data. The FA difference for the nongated data showed higher interindividual variation (i.e., higher standard deviation) than for the gated data.

**Fig 10:**
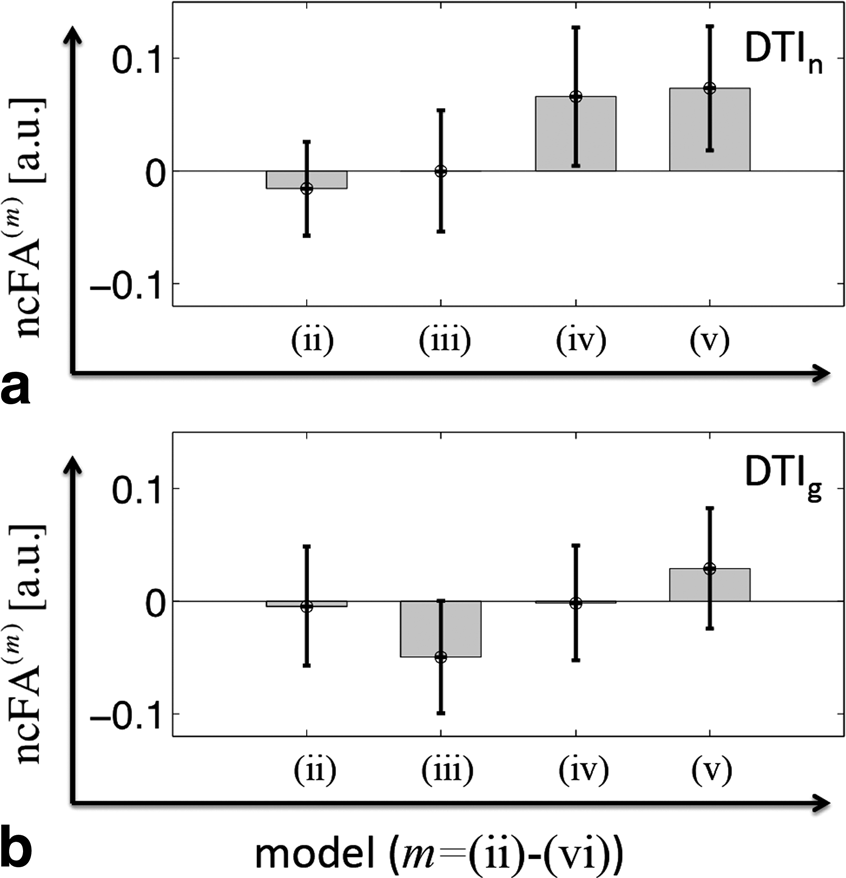
Comparison of FA difference maps obtained from either of the extended tensor models (ii)–(v) with the FA difference obtained from the robust fitting model (vi). For this purpose, the normalized cross-correlation (ncFA^(*m*)^, Eq. [12]) between ΔFA^(vi)^ and ΔFA^(*m*)^ [*m* = (ii)–(v)] was calculated for the DTI_n_ (top row) and DTI_g_ (bottom row) data within the brainstem ROI shown in Figure 1. A slight correlation between the extended tensor model and robust fitting was observed for the nongated data when the extended tensor models with nonlinear regressors [(iv) and (v)] were used. Correlations were not observed for the gated data.

Figure 10 depicts the cross-correlation between FA difference maps obtained from the extended tensor models (ii)–(v) and the robust fitting model (vi). For the nongated data (Fig. 10a), there is a slightly positive correlation between the nonlinear-regressor models (iv) and (v), and the robust fitting model (vi). For the gated data, there is no correlation between the extended tensor models (ii)–(v) and the robust fitting model (vi) (i.e., error bars greater than effect, Fig. 10b), and even a possible anticorrelation might exist between the linear-regressor models (ii) and (iii) and the robust fitting model (vi).

## DISCUSSION

We developed a new method to retrospectively correct for physiological noise in DTI and compared it with the data-driven robust tensor fitting approach (38). The noise correction methods are motivated by the conjecture that, even after perfect retrospective correction of spatial misalignments, artefacts will remain in the diffusion signal, e.g., as a result of local cardiac pulsation. We introduced different noise models and showed that adding physiological noise regressors (in short noise regressors) to the standard linear tensor model resulted in better tensor fits with reduced error for both gated and nongated data. Compared to the robust fitting model, the physiological noise models explained more noise, if gated DTI data were used but not if nongated DTI data were used. Moreover, for the nongated DTI data we found a correlation between the FA bias correction of the robust fitting model and the FA bias correction of a specific set of extended tensor models, which model nonlinear effects.

### Physiological Noise Model for DTI

Cardiac pulsation can lead to severe signal-loss artefacts in the diffusion signal and bias the tensor estimates as shown, e.g., in Refs. 22 and 23. In addition to the signal-loss (i.e., nonlinear signal changes), physiological processes can also lead to small and less obvious changes in the diffusion signal, which we approximated by a linear error term in the diffusion signal. We introduced two types of noise regressors for the extended tensor model, the linear (Eq. [7]) and the nonlinear (Eq. [9]) regressor, which respectively modeled small (linear) and more severe (nonlinear) signal changes. For this purpose, the standard tensor model in DTI [Basser et al. (39)] has been extended in such a way that noise regressors modeling physiological effects in the diffusion signal are added to the original six tensor regressors.

We showed that the tensor fit of the extended tensor model outperformed the standard tensor model fit for both gated and nongated data. The highest noise reduction was achieved by the extended tensor models (ii) and (iii) that used linear regressors, whereas it was lowest for the extended tensor models (iv) and (v) that used nonlinear regressors (Fig. 4). Accounting for both respiratory and cardiac effects together [models (iii) and (v)] did not explain more noise than when respiratory effects were neglected [models (ii) and (iv)]. In keeping with previous studies using fMRI (30,54–56) and DTI (17), we found that the physiological noise was most prominent in the basal brain areas, cerebellum, at the edge of the cortex, and at boundaries between tissue and cerebral spinal fluid, e.g., next to the ventricles.

Physiological artefacts not only increase the noise in the tensor fit but also can bias tensor estimates (15,22,23,57). The improved fits using the extended tensor model and robust fitting suggested that physiological-noise-related bias in FA in the brainstem was between 3 and 5% (Fig. 9).

### Effect of Gating

A diffusion sequence that is triggered to the heartbeat can reduce outliers in the diffusion signal [i.e., signal-loss due to cardiac pulsation (22,23,42)]. Our results supported this observation. We found that the robust fitting model (vi) explained less noise if gated DTI data were used. Presumably, this is due to the fact that it specifically targets large outliers and the gated data were affected by less severe outliers. In contrast to the robust fitting model (vi), all extended tensor models [(ii)–(v)] explained more noise for the gated data. One possible explanation could be that the artifacts in the gated data are better modeled by a linear approximation and thus better described by the extended tensor approach. Another reason why the extended tensor model explained more noise for the gated DTI data could be due to the fact that the tensor-fit error was on average greater in the gated than in the nongated data (Figs. 5 and 6a). The higher tensor-fit error level of the gated data, which in turn is another unexpected finding, could be related to the fact that artefacts (e.g., due to vibration, eddy currents, gradient heating, and signal relaxation) depend on the temporal gap between slice acquisitions. In cardiac gating, the temporal gap between every first slice in each slice acquisition block (acquired during one cardiac cycle) is much longer than for the other slices, disturbing the steady state. Interestingly, the noise level of gated and nongated data became most similar when the extended models (ii) and (iii) with linear regressors were used (Fig. 6).

### Cross-Validation of Extended Tensor Models Using Robust Fitting Model as a Reference

As there is no gold standard reference available for DTI, the results of the data-driven robust fitting model were used as a reference to approximately estimate the bias in the tensor fits. Note that the robust fitting approach has its own limitations such as: (a) it only corrects outliers but not small-amplitude changes in the MR signal [see, e.g., Zwiers (38), Fig. 4], (b) it is unspecific, i.e., corrects for any type of outlier (e.g., subject motions, vibration artefacts, and eddy current effects), and (c) its performance will vary with computational parameters, which were determined empirically for some specific sets of data only. Furthermore, it should be kept in mind that the diffusion tensor approach is generally limited in terms of modeling the underlying anatomical structures, e.g., it cannot appropriately represent crossing or kissing fibre tracts [see, e.g., (58)]. Therefore, it may be difficult to find a gold standard at all.

The robust fitting model accounted for more noise than the extended tensor models when applied on the nongated DTI data (Figs. 4a,c and 6). Similar to the robust fitting model, the nonlinear-regressor extended tensor models [(vi) and (v)] explained less noise in the whole brain (Fig. 4c,d) than in the brainstem and cerebellum (Fig. 4a,b), which is probably more affected by physiological-noise-induced outliers. This finding is in accordance with results from previous DTI studies investigating physiological noise (22,23). Moreover, the FA changes obtained from the nonlinear-regressors models [(iv) and (v)] showed a correlation to those from the robust fitting model when using nongated data (see Figs. 7 and 10a). For the gated data, the linear-regressors extended tensor models [(ii) and (iii)] explained more noise than the other models (Fig. 4b,d). Unexpectedly, the FA change due to the noise correction of the linear-regressors extended tensor models (ii) and (iii) showed an anticorrelation to the results of the robust fitting model (Fig. 10b) most prominently in the white matter (Fig. 8). The differences in FA estimates of robust fitting and the extended tensor models may be due to errors in either method or correcting different sources of noise.

### Potential Applications of Noise Correction in Diffusion MRI

With more time points, i.e., diffusion sensitizing directions, the performance of the noise correction should improve due to the increase in degrees of freedom. We found such a trend for the nongated DTI data (results not shown). Thus, the proposed correction will particularly benefit HARDI-like DTI studies [e.g., (59,60)], where a large number of DW images are acquired. The difference map of the tensor-fit errors (Figs. 2 and 3) could be used to assess DTI data quality for each subject, e.g., in longitudinal and multicentre studies. That information could be incorporated in group statistics, e.g., by removing subjects with particularly strong physiological artefacts from the group analysis.

### Methodological Considerations

The physiological noise correction requires peripheral measurements, which are usually not recorded during DTI scans. However, modern scanners offer the possibility to record peripheral measurements and such methods are well established for fMRI (29).

Although this article discusses the noise correction in the context of the standard second-order diffusion tensor model, the approach can be easily adapted to higher-order tensor models (61).

Note that the presented results cannot be necessarily extrapolated to diffusion MRI data using high *b*-values, because at high *b*-values the SNR is usually reduced and it is known from investigations into fMRI that the performance of physiological noise correction is best at high SNR (29).

To compare the tensor-fit error for the different extended tensor models and the robust fitting model, we had to adjust the tensor-fit error by the degrees of freedom. However, we would like to highlight that our model-adjustments are only simple approximations, since they, e.g., assume a Gaussian distribution of the error. More sophisticated adjustment methods are available (51) but were beyond the scope of this article. Also note that in general, no simple one-to-one relation exists between the tensor-fit error and the bias in the tensor estimates.

The proposed models (see Table 1) should be seen as initial developments, which can be further finessed and complemented by other models. Future work might introduce more principled, physiological noise models and thus improve the performance of the extended tensor model, in a similar way to the development of the physiological noise models in fMRI over the last 15 years [e.g., (29–31,52,55)].

In theory, the extended tensor model can also account for instrumental noise effects such as eddy currents (62,63) or vibration artefacts (19,28,64), using eddy currents distortion parameters (26) or the absolute value of the x diffusion gradient amplitude (|*G*_*x*_|) (28) as noise regressors. However, those noise regressors might not be sufficiently orthogonal with respect to the tensor regressors to improve the fidelity of tensor estimates. It is known that nonorthogonal regressors in general linear models increase the variance of the parameter estimates (51). In the extreme case, if a noise regressor was added to the extended tensor model that is parallel to one of the columns of the design matrix, the solution would become unstable, i.e., the condition number of the extended design matrix can approach infinity [see, e.g., (65,66)]. In simulations (not shown), we observed that an extended tensor model that uses |*G*_*x*_| as a noise regressor will not improve the fidelity of the tensor estimates but bias the tensor estimates, although it might reduce the tensor-fit error. Therefore, the noise model approach will always have the drawback that poorly conditioned regressors might exist which fit anatomical structures that cannot be modeled by the tensor model and thus bias the tensor estimates. We recommend a careful design of the noise models included in the extended tensor model.

## CONCLUSION

In this study, we developed an easy to implement extension of the original tensor model of Basser et al. (39) to account for physiological noise in the DTI data and compared it with an established data-driven robust fitting method (38). We demonstrated that the effect of cardiac-pulsation-induced physiological noise in the diffusion signal is relevant and can be corrected using the extended tensor model. The extended tensor model addresses both large-amplitude outliers and small-amplitude signal-changes unlike the robust fitting method. The approach will facilitate the investigation of physiological noise in DTI by providing a robust linear modeling framework. In turn, the approach will benefit from finessed and novel noise models. Furthermore, the extended tensor fit can be combined with the robust fitting method and other retrospective correction methods such as motion and eddy current distortion [e.g., (26)], vibration [e.g., (19,28)], susceptibility [e.g., (67)], and local perturbation field corrections [e.g., (16)]. The combination of these different approaches is an important step toward providing robust DTI with minimal artefacts.
